# Coexistence of Anal Atresia, Anophthalmia and Intestinal Neuronal Dysplasia Type-A in a Newborn

**Published:** 2015-10-01

**Authors:** Hilal Aydın, Atilla Şenaylı, Fatih Mehmet Kışlal, Dilek Sarıcı, Burhan Köseoğlu, Servet Güreşçi

**Affiliations:** 1 Keçiören Education and Research Hospital, Pediatrics Department, Neonatology Unit, Ankara, Turkey; 2 Yildirim Beyazit University, Pediatric Surgery Department, Ankara, Turkey; 3 Keçiören Education and Research Hospital, Pediatric Surgery Department, Ankara, Turkey; 4 Numune Education and Research Hospital, Pathology Department, Deputy Chief Ankara, Turkey

**Keywords:** Anal atresia, Anophthalmia, Intestinal neuronal dysplasia

## Abstract

We report a patient with anal atresia, anophthalmia and intestinal neuronal dysplasia type A.

## INTRODUCTION

Incidence of anorectal malformations (ARM) is 1% among all anomalies. ARM has been associated with various anomalies like VACTERL (or VATER), cardiac anomalies, and urogenital anomalies etc. [1]. Congenital ocular anomalies (coloboma of iris, microphthalmia, cat-eye syndrome etc.) have been reported with ARM [2]. Coexistence intestinal neuronal dysplasia (IND) is also rare in Cat-Eye Syndrome [2, 3]. ARM with ocular anomaly has not been reported with IND [4]. We herein report a case of ARM associated with anophthalmia and IND type A.

## CASE REPORT

A male newborn, product of consanguineous marriage and weighing 2670g, presented with imperforate anus and anophthalmia (Fig.1). Magnetic resonance imaging proved the patient's anophthalmia. Colostomy was formed before definitive operations for ARM. As a routine procedure, biopsy from colostomy orifice was performed. Pathologic evaluation revealed IND type A (Fig. 2). At one year age, anorectoplasty was performed, and colostomy was closed. After operations, bowel movements and defecation are normal. The boy is on follow-up of ophthalmology as well for ocular problems.

**Figure:**
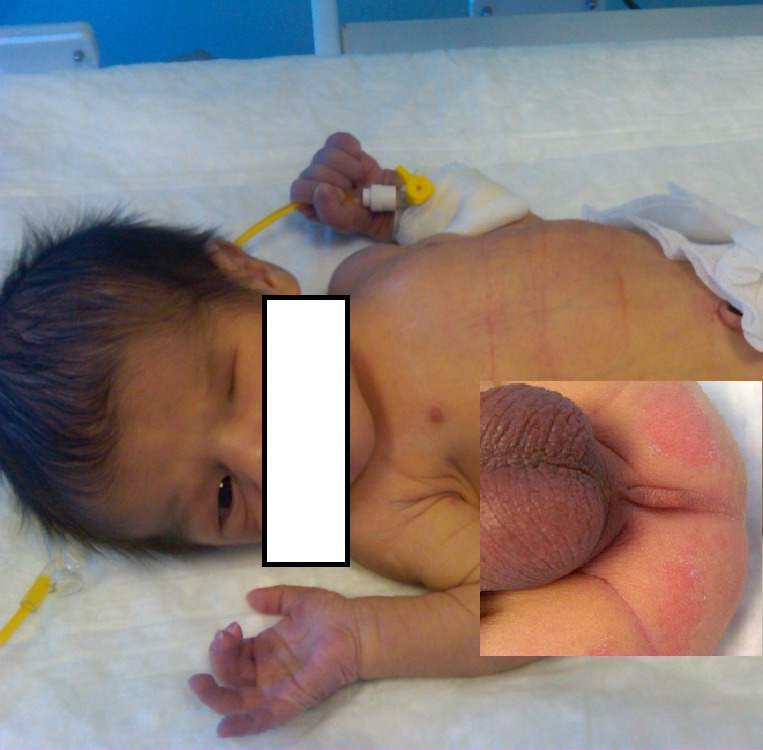
Figure 1: Anophthalmia of the patient. Inset shows imperforate anus.

**Figure:**
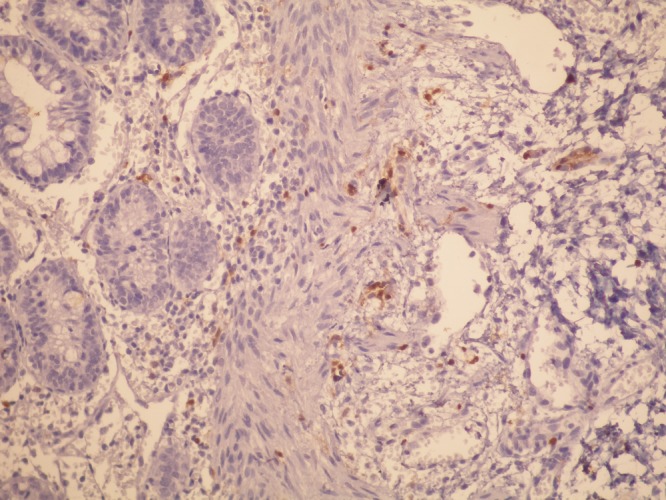
Figure 2: Diminished ganglions and neurons are highlighted by S-100 immunostaining in the submucosa of the colon wall (immunoproxidase x400).

## DISCUSSION

In this report, we presented a case of ARM associated with anophthalmia and intestinal neuronal dysplasia (IND) Type A. Intestinal neuropathies like Hirschsprung’s disease have been reported with ARM. IND is rarely associated with ARM. IND Type A is rare disease, and it is characterized by diminished or absent sympathetic innervations of the myenteric and submucosal plexuses [5]. IND has been reported with Hirschsprung’s disease and infantile pyloric stenosis [4]. Anophthalmia is the absence of the eye. Eighty percent of the anophthalmia cases had other malformations [2, 6]. Neural tube defects, facial clefts and limb reductions are some these malformations [6]. Pathogenesis for these associations is not clear [2]. Coloboma of the iris has been reported with ARM [2]. This association of ARM with anophthalmia and IND has not been reported earlier to the best of our knowledge.

## Footnotes

**Source of Support:** Nil

**Conflict of Interest:** Nil
